# Vigilance and Well-Being in Daily Life: An Examination of Race Differences

**DOI:** 10.1007/s12552-026-09510-3

**Published:** 2026-06-19

**Authors:** Emily C. Noyer, Angela Turkelson, Kira S. Birditt

**Affiliations:** 1 Department of Psychology, University of California, Davis, Davis, USA; 2 Institute for Social Research, University of Michigan–Ann Arbor, Ann Arbor, USA

**Keywords:** Vigilance, Cardiovascular reactivity, Ecological momentary assessment, Emotional well being

## Abstract

The anticipation of discrimination, referred to as vigilance, may be an important driver of racial health inequities in cardiovascular disease and mortality. The current study examined how vigilance is experienced in daily life and its implications for daily cardiovascular health and emotional well-being. Participants included 80 Black and 89 White adults ages 33 to 91, from the Detroit tri-county metropolitan area, who completed ecological momentary assessments (EMAs) via mobile devices regarding experiences of vigilance and emotional well-being every 3 hs, and wore a heart monitor for 4 consecutive days. Data were collected from 2018 to 2020. Multilevel models revealed that Black individuals reported more frequent experiences of vigilance than White individuals, and that vigilance was associated with poorer emotional well-being (higher negative affect; lower positive affect) and higher heart rate in the 3-h periods in which respondents reported vigilance. Race-stratified models demonstrated that vigilance was associated with greater negative affect among Black and White individuals, and lower positive affect among the White individuals only. Vigilance was associated with higher heart rate variability for Black individuals only, indicating greater physiological coping. This adaptation among Black individuals may arise from more frequent experiences of vigilance in daily life and may serve to buffer physiological and emotional effects of daily vigilance. This study demonstrates how vigilance may “get under the skin”, among Black and White individuals, which could pose effects on cardiovascular functioning over the long term.

**Supplementary Information:**

The online version contains supplementary material available at https://doi.org/10.1007/s12552-026-09510-3.

## Introduction

Extreme racial inequities in health exist in the United States with Black individuals having higher rates of chronic diseases and higher mortality rates than White individuals (Benjamins et al., 2017; Wadhera et al., 2021). Theorists postulate that the anticipation of racism and discrimination, a construct referred to as vigilance, is a major contributor to racial health disparities (Phelan & Link, 2015; Williams & Mohammed, 2009). Notably, persistent anticipation of stressful events is tied to the biological stress response and can cause physiological wear and tear on the system (Lazarus, 1999). Vigilance is associated with poorer physical health including higher blood pressure and less arterial vessel elasticity (Clark et al., 2006; Sawyer et al., 2012). Although there have been studies examining the experience of vigilance and its implications for overall health, there is a lack of research on the daily experiences of vigilance among Black and White adults and how those experiences contribute to variations in emotional well-being and cardiovascular health. Therefore, the present study examines vigilance using ecological momentary assessments (EMAs) every 3 hs over 4 consecutive days, and the implications of vigilance for emotional well-being and cardiovascular health using an electrocardiogram monitor (ECG) monitor.

### Theoretical Framework

This study is guided by several theories in the literature including structural racism (Bonilla-Silva, 1997), race socialization (Hughes & Chen, 1997; Lesane-Brown, 2006), vigilance (Clark et al., 2006; Feagin, 1991), and the exposure reactivity model (Almeida, 2005). Structural racism refers to the pervasive practices and policies that exist throughout society and maintain disadvantages towards certain groups of people, namely people of color (Krieger, 2014). The institutions upholding these policies and practices are widespread in society and a racial hierarchy develops in which those who are considered superior have preferential access to societal rewards such as in politics, health, economics, and social situations (Bonilla-Silva, 1997). Those who are considered inferior as the result of structural and systemic racism do not have access to those same resources (Bonilla-Silva, 1997). This gives rise to racial inequalities and discrimination in housing, health, and employment (Phelan & Link, 2015).

Adult variation in reactions to inequalities and discrimination may stem from socialization efforts among marginalized families. Specifically, race socialization among Black families involves the transmission of values, behaviors, and perspectives to develop racial identity, and to prepare children for potential negative race-related interactions based on greater likelihood of experiencing racism that stems from the larger socio-political structure (Fischer & Shaw, 1999; Hughes & Chen, 1997; Lee et al., 2019; Lesane-Brown, 2006). Indeed, daily anticipatory thoughts and behaviors related to the potential of experiencing discrimination, known as vigilance, has been long documented (Bois & Eaton, 1899; Feagin & Sikes, 1994; Sellers et al., 2003), and the subjective perception of racism (e.g., through discrimination) has health implications (Clark et al., 1999).

We focus on daily experiences of vigilance and their implications for daily well-being. The nature of vigilance can continually activate autonomic stress response systems, which over time may create large impacts on health (McEwen, 1998). However, studies to date have examined retrospective reports of vigilance rather than experiences of vigilance in daily life. According to the exposure reactivity model (Almeida, 2005), it is important to examine daily experiences as the build-up of irritations from daily stressors, like vigilance, can generate serious negative implications for emotional and physical well-being through persistent activation of stress responses (Lazarus, 1999).

With previous cross-sectional studies finding that vigilance is linked to physiological reactivity (Clark et al., 2006) and depressive symptoms (Watson-Singleton et al., 2019), experiencing higher than usual vigilance in daily life may therefore be associated with greater emotional and cardiovascular reactivity within the same daily period. However, there is a lack of research examining within-person fluctuations of daily vigilance in association with daily emotional and cardiovascular well-being, which would provide temporally specific and ecologically valid information on vigilance as a potential contributor to health disparities.

### Race Differences in Vigilance

Previous studies have shown that Black individuals experience greater vigilance than White individuals across different samples (Hicken et al., 2013a, 2013b; Hines et al., 2018; LaVeist et al., 2014). Using data from the Chicago Community Adult Health Study, Hicken et al. (2013a, 2013b) found Black adults reported the highest levels of vigilance when compared with White and Hispanic adults. Hicken et al. (2018) found that Black participants were more likely to report experiencing vigilance once a week or more compared to White and Hispanic participants, and the highest levels of vigilance were reported by Black women when compared with White and Hispanic women (Hicken et al., 2018). These studies assessed how often participants experienced vigilance in their general day-to-day life using a scale with four questions given at one point in time. Similarly, a multisite study looking at health disparities in areas where White and Black individuals live together found that Black participants had significantly higher scores of vigilance compared to White participants (LaVeist et al., 2014). In addition, Hines et al. (2018) found that Black participants had a higher mean vigilance score compared to White participants. Taken together, these findings demonstrate the established racial differences in reported vigilance among cross-sectional studies. However, there is a lack of research on the race differences in reported daily vigilance as measured through intensive longitudinal assessments, which could more accurately capture how reports of vigilance vary by race.

### Vigilance and Emotional Well-Being

Vigilance has well-documented associations with greater symptoms of depression and anxiety across populations (Chae et al., 2021). In areas with high racial tension the effects on emotional well-being may be greater. Specifically, a study examining the relationship between police brutality, vigilance, and depressed mood found that symptoms of generalized anxiety and depression were associated with heightened vigilance in Black adults, and that heightened vigilance partially explained the relationship between experienced police brutality and mental health concerns (Alang et al., 2022). Further, the presence of depressive symptoms in Black students at a predominantly White university was positively associated with race-related vigilance (Watson-Singleton et al., 2019). Using The Detroit Area Study data, Himmelstein et al. (2015) assessed cross-sectional vigilance and emotional well-being in Black participants by examining experiences of vigilance, stress, and discrimination over the past month and found that the relationship between discrimination and stress was explained through vigilance coping strategies. Additionally, a laboratory study (Hill & Hoggard, 2018) found that, among Black women, the link between race-related vigilance and depression was mediated by rumination, which is a risk factor for poor mental health (Brosschot et al., 2010). Another study found that the differences in depression prevalence between Black and White adults, where White adults report greater depression than Black adults, was weakened when accounting for vigilance (LaVeist et al., 2014). This indicates that vigilance may contribute to depression (LaVeist et al., 2014). However, more research is needed to examine within-person associations among daily vigilance and well-being.

### Vigilance and Cardiovascular Reactivity

Black individuals experience higher rates of cardiovascular disease and hypertension than White individuals (Benjamins et al., 2017; Wadhera et al., 2021), and vigilance may be an important factor in this link. A study examining vigilance and large arterial elasticity in Black youth (age *M* = 11.5, *SD* = 1.4), which is a sign of endothelial dysfunction and is linked to cardiovascular events, found a negative relationship between baseline large arterial elasticity and racism-related vigilance in Black boys, but not girls (Clark et al., 2006). This indicates that among boys, chronic experiences of racism-related vigilance were associated with cardiovascular dysfunction. Additionally, there was a positive association of large arterial elasticity reactivity during a task and racism-related vigilance among Black boys, suggesting that while chronic perceptions of race-related vigilance are linked to compromised arterial functioning, the increased arterial responsiveness among boys could be adaptive (Clark et al., 2006). This is indicative of how racism “gets under the skin”, thereby minimizing psychological stress associated with racism and favoring impacts to arterial elasticity.

Moreover, cross-sectional studies examining hypertension provide important insight into the link between cardiovascular health and vigilance. For example, Hicken et al. (2013b) found that vigilance was significantly associated with greater hypertension rates for Black individuals but not for White individuals, and that hypertension prevalence disparities between Black individuals and White individuals increased as vigilance rates increased. However, another study found that, among White participants, those who experienced discrimination and reported vigilance had lower odds of hypertension compared to those who did not report vigilance, suggesting a protective effect from vigilance against the negative health outcomes associated with discrimination (Hines et al., 2018). The inconsistent findings regarding vigilance and cardiovascular reactivity demonstrate a need for additional data on within-person links between vigilance and cardiovascular reactivity over time.

### The Present Study

Vigilance, emotional well-being, and cardiovascular health are typically assessed cross-sectionally. This lacks the information needed to determine the within-person associations between vigilance, emotional well-being, and cardiovascular reactivity as they occur in daily life. Thus, the present study uses ecological momentary assessment to address gaps in the literature on the frequency of daily vigilance and its associations with daily emotional well-being and cardiovascular reactivity. We use measures of positive and negative affect to assess momentary emotional well-being, and momentary heart rate (HR) and heart rate variability (HRV) to assess daily cardiovascular well-being. Indeed, negative affect has associations with stress and worse psychological health (Kirkegaard Thomsen, 2006; Watson & Pennebaker, 1989), and heart rate (HR) and heart rate variability (HRV) are associated with daily psychological stress and indicate increased stress through autonomic nervous system activation (Kim et al., 2018; Taelman et al., 2009). Accordingly, this study examines associations among daily vigilance, emotional well-being and two measures of cardiovascular reactivity among Black and White individuals to address several research questions. First, we ask whether there are race differences in the experience of vigilance in daily life, hypothesizing that Black individuals would experience more frequent experiences of daily vigilance compared to White individuals. Second, we ask whether daily vigilance is associated with variations in daily emotional well-being and cardiovascular reactivity, and whether those links vary by race. We hypothesize that greater daily vigilance would be associated with increased negative affect, decreased positive affect and dysregulated cardiovascular stress reactivity (i.e., higher heart rate, lower heart rate variability), and that these effects would be stronger among Black individuals compared to White individuals.

## Method

**Participants** Participants are from the Stress and Well-being in Everyday Life (SWEL) Study who were selected from the second and third waves of the Social Relations Study (SRS; T. Antonucci, PI). The study received approval from the Institutional Review Board at The University of Michigan. All participants completed informed consent and gave their agreement to participate. For additional methodological details see (Birditt et al., 2023).

A total of 238 (129 White, 109 Black; ages 33–93) participated in the SWEL study. Participants completed a baseline interview followed by 4–5 days of EMAs (every 3 h) and were instructed to wear an ECG monitor 24 h a day throughout their daily lives. The SWEL sample was collected by using propensity score matching (Guo & Fraser, 2014) to match eligible Black SWEL participants (*N* = 298) to one or two potential White SWEL participants (*N* = 364) on key characteristics including gender, education, age, social network size, and hypertension status. All participants completed informed consent and gave their agreement to participate. The original aim was to enroll 300 participants (150 White and 150 Black); however, data collection was halted due to the coronavirus 2019 pandemic (COVID-19). With the goal of 300, the SWEL study achieved 238 or 79% of the target sample (109 Black individuals and 129 White individuals).

The sample size varied by the outcome of interest. A total of 238 SWEL participants completed the baseline, and of those 238 participants with a baseline a total of 169 had EMA data on daily vigilance, negative affect, and positive affect. Therefore, 69 participants who completed the baseline did not complete the EMA portion of the study. Comparing the 169 who completed the EMAs portion of the study with the 69 participants who were missing the EMAs, revealed they did not differ on race, gender, marital status, network size, self-rated health, or depressive symptoms. Although, the EMA sample was significantly younger (*b* = − 0.06, *SE* = 0.01, *p* < .001) and had more years of education (*b* = 0.17, *SE* = 0.08, *p* < .05) than those who were missing the EMAs. The 169 participants in the EMA portion of the study completed an average of 17.2 (72%) of the EMAs (69% for Black individuals and 74% for White individuals). The range of completions was 1–26 EMAs and 19.5% of participants had less than 4 days of EMAs, 76.9% had 4 days and 3.6% had 5 days. The total number of EMAs completed was 2905 (Black = 1326, White = 1579). There were five individuals who only completed the EMA portion and were missing heart outcomes, making the sample size for heart outcome 164. The 164 individuals with heart outcomes and EMA data were compared with the five EMA participants who were missing heart outcomes, and there were no significant differences in race, gender, marital status, network size, self-rated health, age, education, or depressive symptoms. To summarize, analyses examining positive and negative affect included 169 participants, and analyses examining HR and HRV included 164 participants.

**Procedure** Participants completed face-to-face baseline interviews in their homes. If participants lived more than 50 miles away or if the interview occurred during the pandemic, the interview was conducted over the phone. The interview was followed by 4–5 days of EMA and ECG data collection. Interviewers demonstrated to the respondent (either by phone or in person) how to complete the daily questionnaires on a provided smartphone and how to wear the BodyGuardian heart monitor. Participants were paid $50 for the baseline assessment, and $150 for completing daily surveys and the ambulatory assessments of stress and biological reactivity (total possible compensation $200).

### Baseline Measures

**Race** Data on race was collected from adult participants in Wave 1 of the SRS. They were asked, “Are you White, Black, Native American, Asian, Hispanic or another race?” Participants were asked, “Which do you feel best describes your race?” if they chose more than one race. If the participant from Wave 1 was a child, race was coded by interviewer observation (*n* = 44). Participants who only completed Wave 3 of the SRS and not Wave 1 were included in our analyses (*n* = 10), and were asked an open-ended question, “What is your ancestry or ethnic origin?” Only participants who identified as White or Black were included in our analysis. White respondents were coded as 0, and Black respondents were coded as 1.

**Covariates** We included age based on birth year (continuous) gender (dichotomous, − 1 = male, 1 = female), years of education (continuous), marital status (dichotomous, − 1 = never married, widowed, or divorced; 1 = living with partner/married), heart trouble (dichotomous, − 1 = no; 1 = yes), and heart medication status (dichotomous, − 1 = no; 1 = yes). Depressive symptoms were evaluated with the eight-item Depression scale from the Center for Epidemiological Studies (*α* = 0.81) (Radloff, 1977).

### Daily Measures

**Vigilance** Individuals indicated whether they did any of the following in the last 3 h: (1) Try to prepare for possible insults from other people before leaving home, (2) Feel that you always have to be very careful about your appearance (to get good service or avoid being harassed), (3) Carefully watch what you say and how you say it, (4) Try to avoid certain social situations and places. These items are adapted from the ethnographically based instrument from David R. Williams, Ph.D, MPH, the Racism-Related Vigilance Scale (Hicken et al., 2013a, 2013b), and has been used to assess the association between vigilance and health outcomes in the Detroit Area study and the Chicago study (Clark et al., 2006; Hicken et al., 2013a; Himmelstein et al., 2015). Respondents were asked to check all that applied, and 3-h periods in which at least one experience of vigilance occurred were coded as 1 vigilance occurred or 0 no vigilance occurred. An additional analysis examining the association between a baseline measure of vigilance to the daily measure of vigilance is included in the post hoc analyses section as evidence of validity for the daily measure.

**Negative Affect** Adapted from the Positive and Negative Affect Schedule scale (Watson et al., 1988) and included the extent to which respondents experienced the following negative emotions in the last 3 h: tired, worried/anxious, tense/stressed, irritated, lonely, angry, bored, and sad using the following scale: (1) Not at all, (2) A little, (3) Somewhat, (4) Quite a bit, and (5) A great deal. Items were averaged to create a negative affect score for each 3-h period (*α* = .76).

**Positive Affect** Adapted from the Positive and Negative Affect Schedule scale (Watson et al., 1988) and included the extent to which respondents experienced the following positive emotions in the last 3 h: energetic, loved, happy, calm, content, excited, proud, and optimistic using the following scale: (1) Not at all, (2) A little, (3) Somewhat, (4) Quite a bit, and (5) A great deal. Items were averaged to create a positive affect score for each 3-h period (*α* = .85).

**Heart Rate** The BodyGuardian heart monitor is a portable electrocardiogram (ECG) monitor that is worn by participants on their chest for 24 h a day. The specific model is the BodyGuardian MINI by Boston Scientific Cardiac Diagnostics, Inc. The monitor transmitted data via Bluetooth connection to a cellphone which then transmitted data to a secure server. The BodyGuardian heart monitor detected the ECG signals at 256 Hz sample rate. Artifacts due to technical or physiological issues in the ECG signal were identified by the BeatLogic algorithm. Additionally, we removed beat intervals following artifacts from the HRV and HR calculations.

The monitor vendor provides measures of HR. HR is derived by detecting the R wave component of the QRS complex for both normal and premature ventricular complexes (PVCs). The system calculates the interval between R waves (R–R interval) and processes this information to derive an average HR value every 10 s. We created an average HR for each 3-h period and removed raw HR values of less than 20. Furthermore, the BodyGuardian ECG monitor has been demonstrated as comparable to in-clinic measures of HR, as adequate for continuous HR monitoring (Izmailova et al., 2019), and valid for beat detection sensitivity and positive predictive value as determined by the FDA-recognized consensus standard (Teplitzky et al., 2020).

**Heart Rate Variability** To measure cardiovascular reactivity, the study calculated HRV as a time-domain index root mean squared successive differences (RMSSD). We calculated each successive time difference between heartbeats, averaged the result, and then squared each value to obtain a 10-s interval for RMSSD.

Averages scores of RMSSD were calculated by aggregating the 10-s RMSSD values across 3-h interval time stamps that correspond to surveys assessing vigilance. To obtain a normal distribution, we log-transformed average scores of RMSSD at each 3-h interval (LnRMSSD), which is an applicable and reliable measure for daily HRV measurement (Schwerdtfeger et al., 2020).

### Analysis Strategy

A total of 2491 surveys were used in this analysis. All surveys had negative affect and positive affect, and 2382 surveys had HR and HRV data from the corresponding 3-h period. We first analyzed means (*M*) and standard deviations (*SD*), followed by *t*-tests and chi-squared tests to assess whether there were significant race differences in covariates, negative affect, positive affect, HR and HRV. Next, variance for measures of vigilance was analyzed to ensure sufficient variability. Between person variance for summed vigilance was 66% and within person variance was 34%. Between person variance for dichotomous vigilance was 64% and within person variance was 36%. Next, we estimated multilevel logistic models in R Studio (R Core Team, 2024) to examine first whether there were race differences in vigilance. The model included two levels in which the EMAs were nested in participants. Race was the predictor and the outcome was the dichotomous vigilance score. The models predicting vigilance controlled for age, education, gender, marital status, and depressive symptoms.

Next, to examine the effects of daily vigilance on daily emotional well-being and cardiovascular reactivity we used the dichotomous vigilance score as a predictor of four outcomes that were assessed every 3 h: negative affect, positive affect, mean HR, and HRV. The models predicting negative affect or positive affect controlled for age, gender, education, marital status, and depressive symptoms. We also controlled for the person-level proportion of every 3-h periods in which participants experienced vigilance to separate the within- and between-person effects of vigilance. The models predicting mean HR and HRV controlled for heart problems in addition to the aforementioned covariates. We did not include heart medication as a covariate in models because it was highly correlated with heart trouble (*r* = 0.68).

Next, we used stratified models between Black and White individuals to examine if the associations between daily vigilance and the outcomes varied by race. Covariates for the corresponding outcomes were included in the models. Furthermore, post-hoc analyses were conducted to test the robustness of our findings against the inclusion of additional covariates known to impact cardiac outcomes (See Supplementary Material).

## Results

### Descriptives

An examination of means revealed that there were no significant differences in negative affect, positive affect, age, gender, education, self-rated health, marital status, and depressive symptoms between Black and White individuals (Table 1). There were significant differences between Black (*M* = 85.43, *SD* = 13.43) and White individuals (*M* = 83.85, *SD* = 13.78; *t*(2712.2) = − 3.06, *p* = .002) for HR, and between Black (*M* = 3.34, *SD* = 0.60) and White individuals (*M* = 3.55, *SD* = 0.69; *t*(2721.7) = 8.57, *p* < .001) for HRV (Table 1).

**Table 1:** Sample Descriptives

Variable	Black (*n* = 80)			White (*n* = 89)			Sig diff
	*M*	*SD*	Range	*M*	*SD*	Range	
Age	52.01	11.46	33–91	53.62	13.9	33–91	ns
Education	14.01	1.8	10–17	14.74	1.91	10–17	ns
Income	7.19	2.81	1–13	8.93	2.73	1–13	ns
Self-rated health	3.61	0.89	1–5	3.83	0.97	1–5	ns
Depressive symptoms	6.99	4.96	0–22	5.37	4.64	0–22	ns
Vigilance	7.34	6.84	0–22	2.49	4	0–22	**
Negative affect	1.34	0.50	0–8	1.37	0.46	0–8	ns
Positive affect	3.12	0.82	0–8	3.07	0.74	0–8	ns
Heart rate	85.43	13.43		83.85	13.78		*
Heart rate variability	3.34	0.60		3.55	0.69		**

	%	*n*		%	*n*		Sig diff
Female	68.8	55		64	57		ns
Marital status	48.8	39		60.7	54		ns
Heart medication	8.8	7		5	4		ns
Heart problems	11.3	9		6.7	6		ns

Vigilance descriptives reflect total EMA observations

*Sig diff* significant difference, *SD* standard deviation, *SE* standard error, *ns* non-significant

*p < .01, **p < .001

**Fig. 1:**
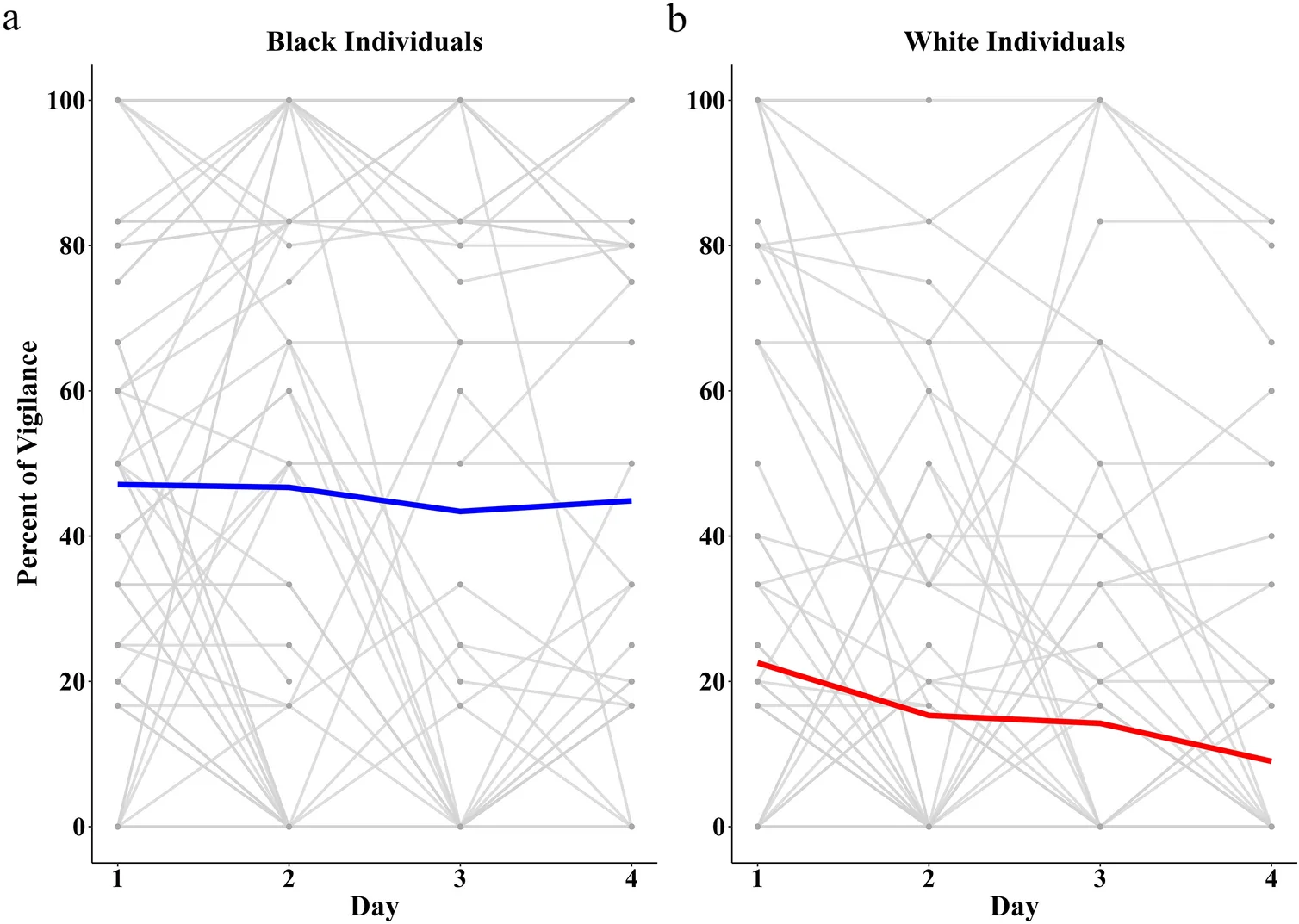
Vigilance in Every 3-h Period by Race. *Note* Daily vigilance average for Black participants on the left panel with a blue, solid line representing the daily average of vigilance (**a**). Daily vigilance average for White participants on the right panel with a red, solid line representing the daily average of vigilance (**b**). Each light grey line represents a respondent (color figure online)

Examining the average daily percent of vigilance, we found that Black participants had a higher average of reported vigilance (51.04%) compared to White participants (16.6%) (Fig. 1). The average across all participants was 33.82%.

A total of 113 (66.9%) participants reported experiencing vigilance in their daily lives at least once over the 4–5 days. The response, “Carefully watch what you say and how you say it” was the most common aspect of vigilance experienced across Black and White individuals with 103 EMA surveys (60.9%). Participants reported an average of 4.8 3-h periods of vigilance with a range from 0 to 22.

### Are There Race Differences in the Experience of Vigilance in Daily Life?

A model examining the association between race and daily vigilance revealed that Black individuals reported experiencing vigilance more often than White individuals (OR 18.28, 95% CI 6.06–55.10; Table 2).

**Table 2:** Logistic Regression Examining Daily Vigilance as a Function of Race

Variable	Vigilance	
	*OR*	*SE*
Black	18.28***	0.56
Age	0.99	0.02
Female	0.84	0.57
Education	0.47***	0.15
Marital status	0.41	0.56
Depressive symptoms	1.22***	0.06

*OR* odds ratio, *SE* standard error

***p < .001

### Are There Implications for Daily Vigilance on Emotional Well-Being and Cardiovascular Reactivity in Daily Life?

A model examining the within-person association between daily vigilance and negative affect across the full sample revealed that individuals who experienced vigilance also reported greater negative affect in the same 3-h period (*b* = 0.10, SE = 0.01, *p* < .001; Table 3). Next, a model examining associations between daily vigilance and positive affect revealed that individuals who experienced vigilance reported lower positive affect in the same 3-h period (*b* = − 0.06, *SE* = 0.02, *p* < .001; Table 3).

**Table 3:** Multilevel Models Examining Daily Affect as a Function of Vigilance in the Full Sample

Variable	Negative affect		Positive affect	
	*b*	*SE*	*b*	*SE*
Within-person vigilance	0.10***	0.01	− 0.06***	0.02
Between-person vigilance	0.00*	0.00	0.00*	0.00
Age	− 0.01***	0.00	0.01	0.00
Female	− 0.02	0.05	0.17	0.10
Education	0.01	0.01	− 0.02	0.03
Marital status	− 0.12*	0.05	0.00	0.10
Depressive symptoms	0.02***	0.01	− 0.05***	0.01
− 2 log likelihood	2403.27		3821.74	

Within-person vigilance refers to fluctuations in an individual’s vigilance relative to their own average, whereas between-person vigilance refers to differences in average vigilance between individuals. Both were included in the same model

*SE* standard error

*p < .05, ***p < .001

A model examining the within-person association between daily vigilance and mean HR revealed that individuals who experienced vigilance also experienced higher mean HR in the same 3-h period (*b* = 0.74, *SE* = 0.33, *p* < .05; Table 4). The model examining the association between daily vigilance and HRV was not significant (*b* = .01, *SE* = .01, *p* = .34; Table 4).

**Table 4:** Multilevel Models Examining Mean Heart Rate and Heart Rate Variability as a Function of Vigilance in the Full Sample

Variable	Mean heart rate		Heart rate variability	
	*b*	*SE*	*b*	*SE*
Within-person vigilance	0.74*	0.33	0.01	0.01
Between-person vigilance	− 0.03	0.02	0.00	0.00
Age	− 0.18**	0.06	0.01*	0.00
Female	2.41	1.52	− 0.04	0.09
Education	− 0.57	0.43	0.02	0.03
Marital status	3.00*	1.52	− 0.08	0.09
Depressive symptoms	0.57**	0.17	− 0.02	0.01
Heart trouble	− 7.36**	2.62	0.44**	0.16
− 2 log likelihood	17,636.59	1736.16		

*SE* standard error

*p < .05, **p < .01

### Is Daily Vigilance Associated with Variations in Emotional Well-Being and Cardiovascular Health in Daily Life Among Black and White Individuals?

**Fig. 2:**
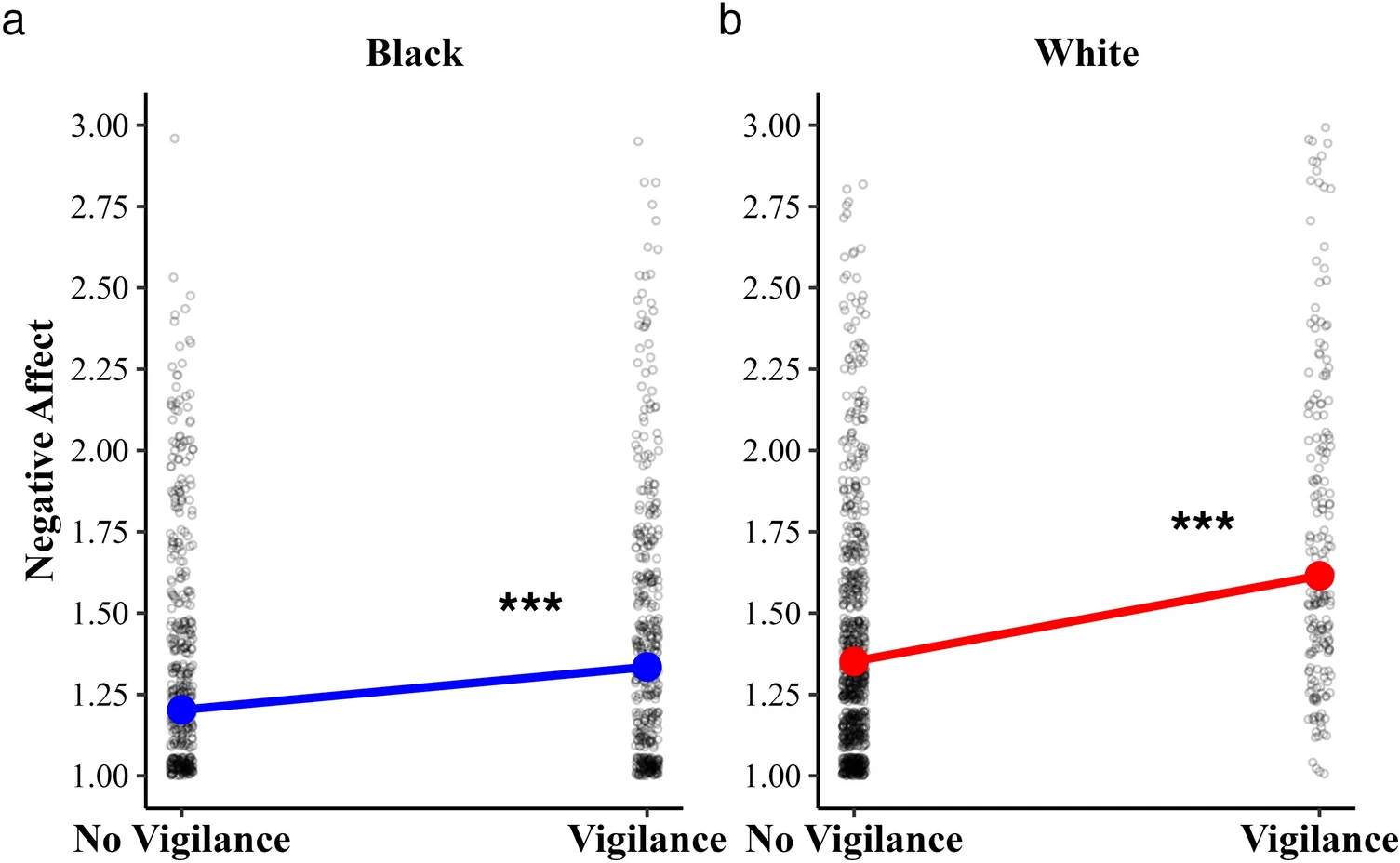
Models Examining Negative Affect as a Function of Vigilance. *Note* ***p < .001. Estimates for negative affect in the presence of vigilance versus no vigilance overlaid on observed values of negative affect for Black participants on the left panel (**a**), and for White participants on the right panel (**b**)

**Fig. 3:**
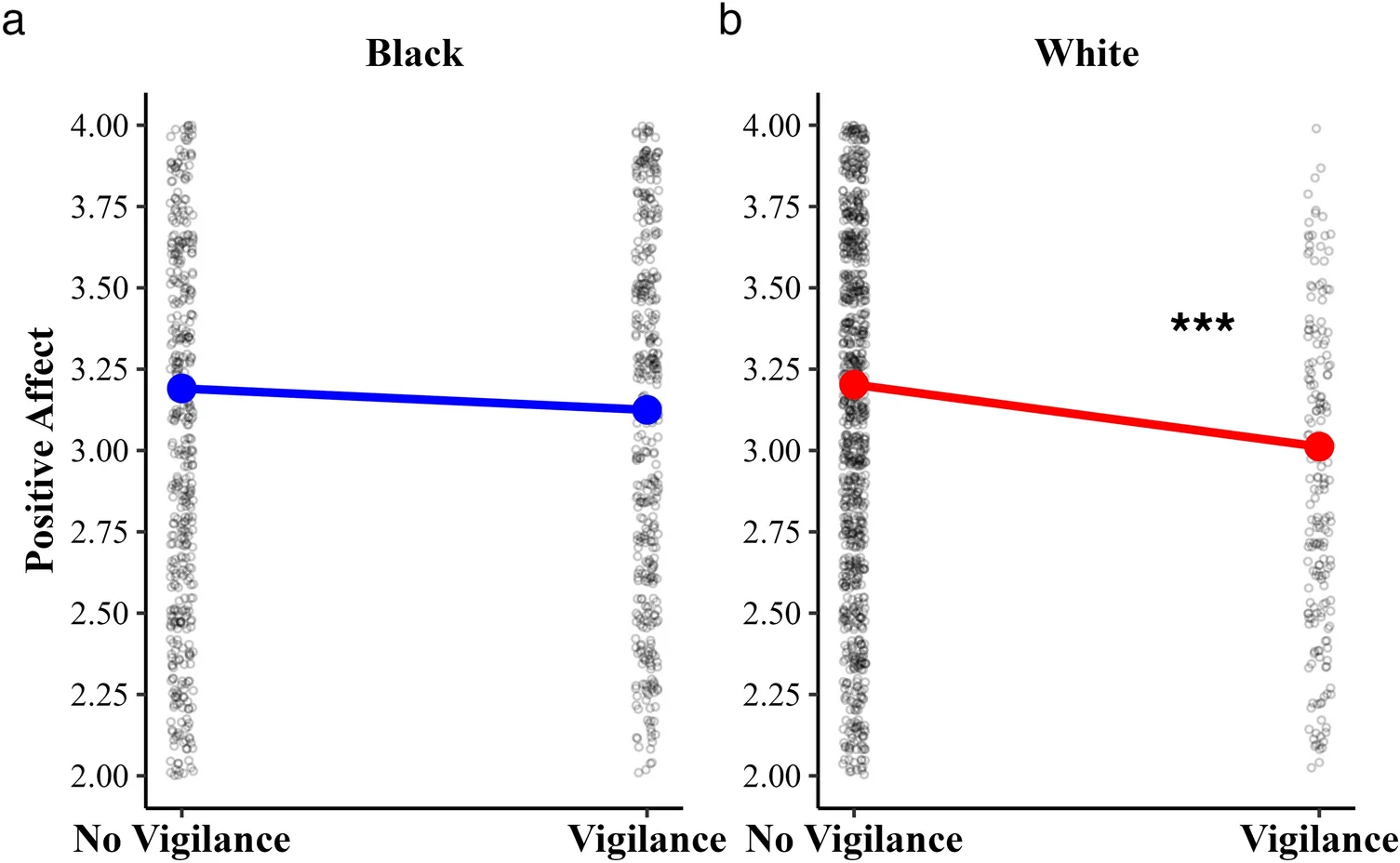
Models Examining Positive Affect as a Function of Vigilance. *Note* ***p < .001. Estimates for positive affect in the presence of vigilance versus no vigilance overlaid on observed values of positive affect for Black participants on the left panel (**a**), and for White participants on the right panel (**b**)

We estimated race-stratified models and found a significant within-person association between daily vigilance and higher negative affect among Black individuals (*b* = 0.07, *SE* = 0.02, *p* < .001; Table 5; Fig. 2) and White individuals (*b* = 0.13, *SE* = 0.02, *p* < .001; Table 5; Fig. 2). The model examining positive affect found a significant within-person association between daily vigilance and lower positive affect among White individuals (*b* = − 0.10, SE = 0.02, *p* < .001; Table 5; Fig. 3), but the association was not significant for Black individuals (*b* = − 0.03, SE = 0.02, *p* = .19; Table 5; Fig. 3).

**Table 5:** Multilevel Models Examining Daily Affect Among Black and White Individuals as a Function of Vigilance

Variable	Black				White			
	Negative affect		Positive affect		Negative affect		Positive affect	
	*b*	*SE*	*b*	*SE*	*b*	*SE*	*b*	*SE*
Within-person vigilance	0.07***	0.02	− 0.03	0.02	0.13***	0.02	− 0.10***	0.02
Between-person vigilance	− 0.00	0.00	0.00	0.00	0.00	0.00	0.00	0.00
Age	− 0.01*	0.00	0.01	0.01	− 0.01**	0.00	0.00	0.00
Female	0.01	0.08	0.08	0.17	− 0.03	0.06	0.22	0.13
Education	0.00	0.02	− 0.00	0.05	0.01	0.02	− 0.04	0.04
Marital status	− 0.17*	0.08	− 0.01	0.16	− 0.08	0.06	0.02	0.14
Depressive symptoms	0.02*	0.01	− 0.03***	0.02	0.03***	0.01	− 0.06***	0.02
− 2 log likelihood	1267.20		1883.58		1211.96	2011.68		

*SE* standard error

*p < .05, **p < .01, ***p < .001

**Fig. 4:**
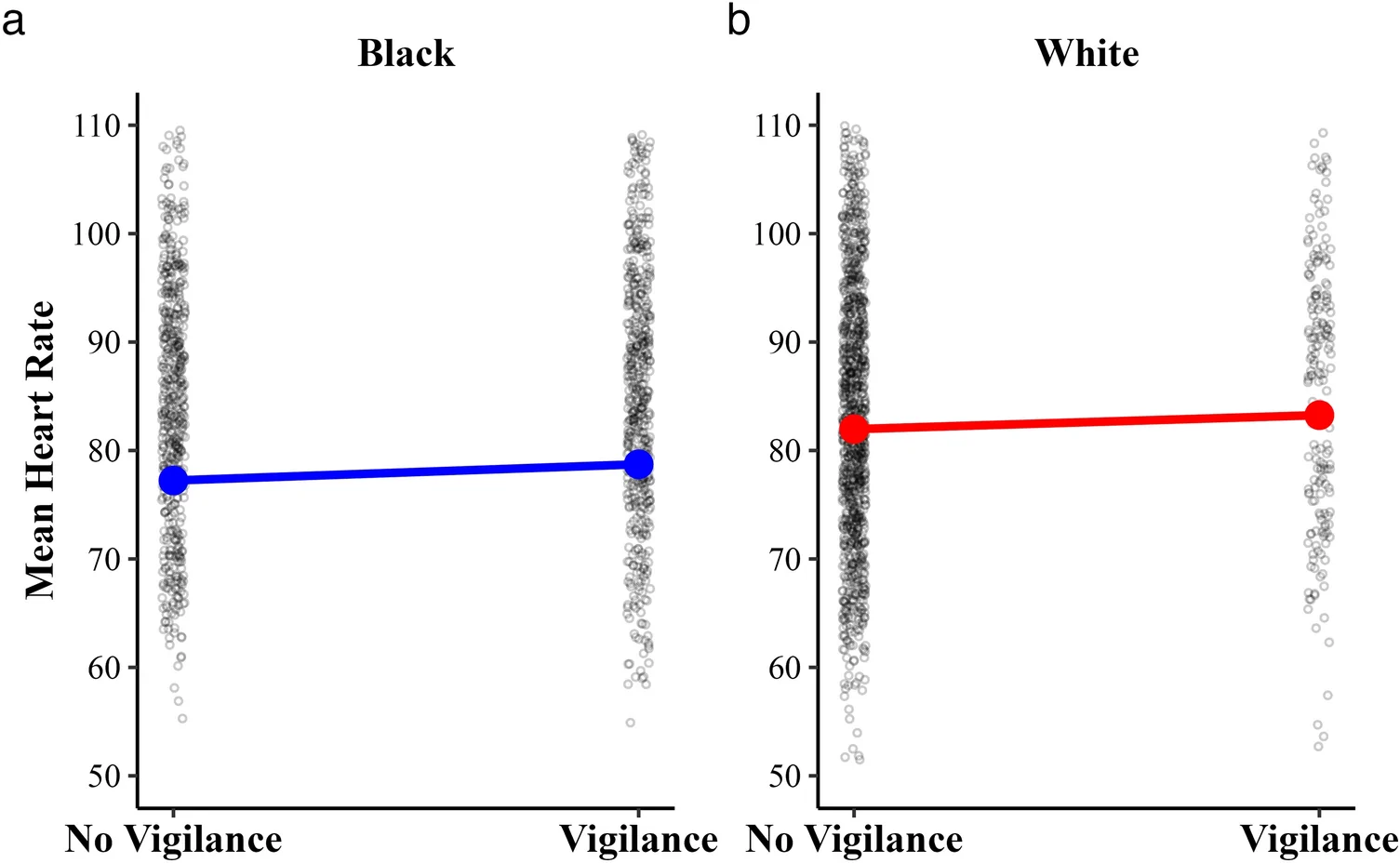
Models Examining Average Heart Rate as a Function of Vigilance. *Note* Estimates for mean heart rate in the presence of vigilance versus no vigilance overlaid on observed values of mean heart rate for Black participants on the left panel (**a**), and for White participants on the right panel (**b**)

**Fig. 5:**
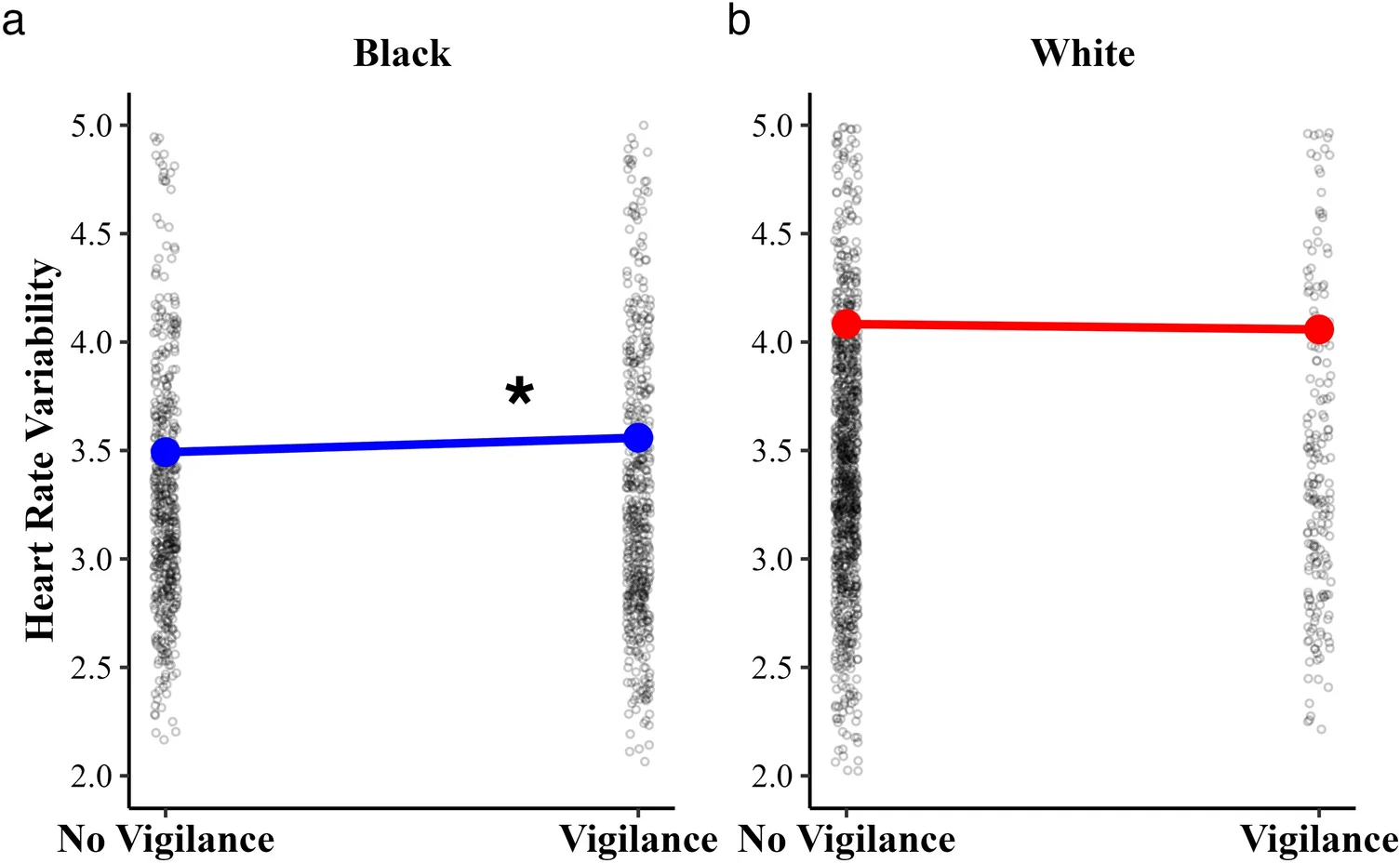
Models Examining Heart Rate Variability as a Function of Vigilance. *Note* *p < .05. Estimates for heart rate variability in the presence of vigilance versus no vigilance overlaid on observed values of heart rate variability for Black participants on the left panel (**a**), and for White participants on the right panel (**b**)

The model examining the within-person association between daily vigilance and HR was not significant for Black (*b* = 0.75, *SE* = 0.42, *p* = .07; Table 6; Fig. 4) or White (*b* = 0.65, *SE* = 0.44, *p* = .15; Table 6; Fig. 4) individuals. The model examining HRV found a within-person association for Black individuals (*b* = 0.03, *SE* = 0.02, *p* < .05; Table 6; Fig. 5), but not for White individuals (*b* = − 0.01, *SE* = 0.02, *p* = .42; Table 6; Fig. 5).

**Table 6:** Multilevel Models Examining Mean Heart Rate and Heart Rate Variability among White and Black Individuals as a Function of Vigilance

Variable	Black				White			
	Mean heart rate		Heart rate variability		Mean heart rate		Heart rate variability	
	*b*	*SE*	*b*	*SE*	*b*	*SE*	*b*	*SE*
Within-person vigilance	0.75	0.42	0.03*	0.02	0.65	0.44	− 0.01	0.02
Between-person vigilance	− 0.07	0.03	0.00	0.00	− 0.01	0.04	0.00	0.00
Age	− 0.08	0.11	0.00	0.01	− 0.20*	0.08	0.01*	0.00
Female	0.48	2.49	0.07	0.13	3.54	2.14	− 0.05	0.13
Education	− 0.90	0.73	− 0.04	0.04	− 0.20	0.60	0.05	0.03
Marital status	1.32	2.36	− 0.15	0.13	4.46	2.25	− 0.08	0.13
Depressive symptoms	0.75**	0.26	− 0.03	0.01	0.65*	0.26	− 0.02	0.02
Heart trouble	− 9.20*	3.58	0.28	0.19	− 6.45	4.48	0.70**	0.26
− 2 log likelihood	1897.96		910.97		9284.45	840.36		

*SE* standard error

*p < .05, **p < .01

### Post Hoc Analyses

See the Supplementary Material for post hoc analyses that examined the slopes between Black and White individuals in an interaction analysis, tested removal of low reliability HR data, examined associations between the baseline vigilance measure and the daily vigilance measure as evidence for validity, and examined the association between cardiovascular measures and vigilance with the addition of covariates known to impact cardiac metrics.

## Discussion

The study aimed to understand daily experiences of vigilance among Black and White adults and the implications of daily vigilance for daily emotional and cardiovascular reactivity. Overall, we found that Black adults were more likely to experience vigilance in daily life than White adults. Moreover, vigilance predicted worse emotional and cardiovascular well-being and these links varied by race.

### Experiences of Vigilance by Race

The present study examined EMA reports of vigilance across 4–5 consecutive days and showed that Black individuals were eighteen times more likely to report vigilance compared to White individuals. Structural racism posits that inequalities exist between Black and White individuals, and these inequalities stem from pervasive policies and practices that uphold discrimination towards Black individuals in politics, health, economics, and social situations. Consequently, this leads to differences in social standing and access to resources, where Black individuals may not have access to the resources to acquire similar social standing as White individuals (Bonilla-Silva, 1997; Krieger, 2014). Therefore, structural racism perpetrates greater experiences of discrimination and racism among Black individuals (Bleich et al., 2019). Methods of navigating negative race-related experiences are transmitted among families to prepare children for potential encounters (Fischer & Shaw, 1999; Hughes & Chen, 1997; Lesane-Brown, 2006), and it has been shown that Black individuals prepare for potential discrimination through changes in thought and behavior (Clark et al., 2006; Feagin, 1991). The current study provides further evidence that Black individuals prepare for potential discrimination through vigilance and is consistent with previous research showing that Black individuals experience greater vigilance than White individuals due to greater experiences of discrimination and racism across the life course (Himmelstein et al., 2015).

### Vigilance and Emotional Reactivity Among Black and White Individuals

The study found that daily affect varied as a function of vigilance, and these associations varied by race. When respondents reported vigilance in a 3-h period they were more likely to report increased negative affect and decreased positive affect in that same 3-h period. Increased negative affect has been linked to poor psychological health, rumination, and stress (Kirkegaard Thomsen, 2006; Watson & Pennebaker, 1989). Race-stratified models indicated similar emotional associations to daily vigilance, with Black and White individuals reporting greater negative affect, and White individuals also reporting lower positive affect in the same 3-h period in which vigilance was reported.

Previous research indicates that Black individuals are at greater risk for experiencing negative emotional well-being outcomes associated with vigilance (Alang et al., 2022; Chae et al., 2021; Feagin, 1991; Himmelstein et al., 2015). Interestingly, our analyses found that White individuals showed similar emotional associations as Black individuals, with greater negative affect. Therefore, the experience of vigilance, for Black and White individuals, is salient enough to be associated with negative emotional states regardless of the frequency of the experience. However, because Black individuals experience more daily vigilance, they are also at risk for experiencing more persistent negative affect in daily life. Increases in negative affect in daily life are associated with suppression and rumination, which are linked to psychopathology (Brans et al., 2013). Indeed, LaVeist et al. (2014) found that vigilance predicted greater odds of experiencing depression, Watson-Singleton et al. (2019) demonstrated that greater vigilance was related to greater depressive symptoms, and Chae et al. (2021) found positive associations between vigilance, anxiety, and depression. This suggests that greater amounts of daily vigilance may be a risk factor for poor mental health outcomes among Black individuals.

In addition, the finding that daily vigilance was associated with a decrease in positive affect among White individuals, but not Black individuals is consistent with research showing that, compared to White individuals, Black individuals tend to maintain higher levels of positive affect while experiencing higher negative affect (Lankarani & Assari, 2017). This could buffer some of the negative psychological outcomes associated with negative affect (Fredrickson et al., 2000).

Black respondents may not show the same reductions in positive affect as White individuals due to more frequent experiences of vigilance as a result of systematic racism. Furthermore, race socialization among Black families prepares children to anticipate negative race-related interactions (Hughes & Chen, 1997; Lesane-Brown, 2006). Thus, Black adults may carry the expectation that vigilance is embedded into daily life and have developed coping strategies that mitigate influences on positive affectivity (Brownlow, 2023; Fischer & Shaw, 1999; Womack & Sloan, 2017). Therefore, these findings may be due to race differences in appraisal of vigilance and greater psychological resilience to vigilance (Almeida, 2005). However, given the persistent and effortful nature of these coping strategies, they have implications for health and racial health disparities (Brownlow, 2023).

### Vigilance and Cardiovascular Reactivity Among Black and White Individuals

The study found that daily cardiovascular reactivity varied as a function of vigilance, and these associations varied between racial groups. Experiencing vigilance within a 3-h period was associated with greater HR in the same 3-h period in the full sample and indicates that the effects from vigilance may act through physiological arousal. This is consistent with literature showing associations between acute daily stressors and cardiovascular reactivity (Vaessen et al., 2021), and supports research and theory that posits vigilance as a stressor (Clark et al., 2006; Sawyer et al., 2012). Indeed, the link between stress and negative health outcomes accumulates over time from daily stressors such as vigilance (Almeida, 2005), and frequent cardiovascular reactivity to a stressor has been associated with hypertension, atherosclerosis, and mortality from cardiovascular disease (Whittaker et al., 2021). Given that Black individuals reported more frequent experiences of vigilance, this may place Black individuals at greater risk for negative health outcomes associated with frequent cardiovascular reactivity.

However, vigilance also predicted greater HRV among Black individuals, but not among White individuals. Literature states that higher HRV indicates greater emotional regulation and self-regulatory behavior in stressful situations (Balzarotti et al., 2017; Thayer et al., 2012). Thus, individuals may show heightened HR due to stress and increased HRV in attempts to regulate that response, which may be an adaptive response to acute stress (Thayer & Brosschot, 2005; Vrijkotte et al., 2000). In this context, higher HRV may serve to adaptively manage the effects of increased reactivity associated with daily vigilance.

Collectively, the current study’s findings of higher HR and higher HRV in relation to daily vigilance illustrate both potential negative and positive cardiovascular implications of daily vigilance. On one hand, greater cardiovascular regulation associated with vigilance could help mitigate risk for cardiovascular disease (Thayer et al., 2010). On the other hand, increased cardiovascular reactivity from vigilance may increase risk for negative health outcomes over time (Almeida, 2005). This interpretation is congruent with previous experimental work demonstrating that higher baseline vigilance among Black boys is associated with increased arterial reactivity during a task, which is considered adaptive in the short-term, as well as reduced arterial elasticity, a maladaptive marker of cumulative cardiovascular activation (Clark et al., 2006). In addition, research on cumulative life stressors and daily stress processes, demonstrate that Black individuals and those who experienced greater stress across the life course were more likely to use coping strategies associated with higher momentary HR (Birditt et al., 2023). Thus, these patterns may reflect the development of physiological responses that stem from greater lifetime stressors, including structural racism.

Although our HRV findings are in contrast with our hypothesis (that Black individuals would show a dysregulated response through higher HR and *lower* HRV), the results support the exposure reactivity model (Almeida, 2005), where frequent exposure to daily stressors, like vigilance, can generate negative impacts for physiological processes through persistent activation. Furthermore, the potential negative and positive implications of these findings may help to explain previous mixed results on cardiovascular health and vigilance, with Hines et al. (2018) showing no association between hypertension and vigilance among Black and White participants living in racially integrated contexts, and Hicken et al. (2013b) showing that vigilance predicted greater hypertension prevalence among Black respondents. Taken together, the current study demonstrates how vigilance ‘gets under the skin’ and impacts the cardiovascular system through potentially adaptive and maladaptive cardiovascular processes. This further demonstrates the need for investigations on cardiovascular health and vigilance as a potential contributor to health disparities.

### Limitations, Future Directions, and Strengths

This study includes limitations that should be addressed in future research. First, we did not include data on lifetime experiences or current experiences of discrimination to understand race differences in vigilance. Furthermore, we do not have data on neighborhoods to understand structural racism, or data on race socialization experiences that may explain variations in vigilance and reactivity. Our EMA data offers a snapshot of 4 to 5 days of life and may not reflect the diversity of experiences that people have and or typical daily experiences, had we included more days or a burst design. Moreover, HR and HRV are only a few factors that contribute to cardiovascular health. Collecting data on daily vigilance and blood pressure would help clarify the link between vigilance and cardiovascular health. Regarding the link between HR and within-person vigilance among Black individuals, the finding was marginal and was comparable to the estimate found for the full sample, indicating that the marginal finding may reflect limited power. Thus, our study would have benefitted from a larger sample size. Further, individual differences in personality and coping resources may affect daily outcomes related to vigilance. For instance, Hill and Hoggard (2018) showed that individuals with higher active coping displayed increased rumination, which mediated the association between race-related vigilance and depressive symptomatology.

In addition, we did not ask participants the reason they felt vigilance, and therefore it is not known whether vigilance was race-related or due to other factors such as age, gender, or disability. The vigilance measure was developed through ethnographic work on Black Americans and may not capture all aspects of vigilance (Clark et al., 2006; Feagin & Sikes, 1994). This was the first time we have adapted vigilance to be used in an every 3-h survey, future research may need to consider more nuanced items to understand feelings of vigilance on a daily basis. Lastly, our participant sample consists of those who resided in the Detroit tri-county area, future research should assess daily vigilance, emotional well-being and cardiovascular health from samples in other geographic regions.

The current study includes numerous strengths. Namely, our use of EMA to assess feelings of vigilance every 3 h during the day for 4 consecutive days addresses gaps in the current literature on the race differences in frequency of vigilance in daily life. Moreover, in comparison to assessing vigilance cross-sectionally, examining vigilance multiple times a day provides greater accuracy on the frequency of vigilance and its relationship to emotional well-being, and cardiovascular reactivity. Notably, no previous studies to our knowledge have assessed the within-person associations between daily vigilance, HR, and HRV. These results contribute novel findings and highlight the importance of incorporating physiological outcomes in future research.

Overall, the current study makes significant contributions to the understanding of vigilance using EMAs and ECG data collected across consecutive days. This methodology allows for the unique study of within-person associations of daily vigilance, race, emotional well-being, and cardiovascular reactivity. Using this approach, we found associations between daily vigilance and reduced emotional well-being through increased negative affect and decreased positive affect, and greater cardiovascular reactivity through higher HR in the entire sample. The race-stratified models examining emotional well-being demonstrated that Black individuals showed increased negative affect in association with daily vigilance, while White individuals showed increased negative affect and reduced positive affect in association with vigilance. Despite the adverse HR and affect associations, daily vigilance was also associated with higher HRV among Black individuals, potentially reflecting physiological coping or regulation due to more frequent exposure to discrimination among Black individuals. Thus, these physiological processes associated with vigilance have the potential to both mitigate and increase risk for negative health outcomes and may explain the mixed findings for the association between vigilance and cardiovascular outcomes.

## Supplementary Information

Below is the link to the electronic supplementary material.

Supplementary file1 (DOCX 1594 KB)

## Data Availability

The data that support the findings of this study are not publicly available due to ethical and privacy restrictions. In accordance with the approved Institutional Review Board protocol and the informed consent process, we agreed to maintain the confidentiality of all study participants. Identifiable information is stored securely and cannot be shared outside the research team.
