# Supplementary material for: Vigilance and Well-Being in Daily Life: An Examination of Race Differences
Source: Race Soc Probl. 2026 Jun 19;18(3):46. doi: 10.1007/s12552-026-09510-3 (PMC13282204; doi:10.1007/s12552-026-09510-3)
Supplement: Supplementary file 1 — Supplementary file1 (DOCX 1594 KB) [file 12552_2026_9510_MOESM1_ESM.docx]

**Supplementary Material**

**Post Hoc Analyses**

The following analyses examined the slopes between Black and White individuals, tested removal of low reliability HR data, examined associations between the baseline vigilance measure and the daily vigilance measure as evidence for validity, and examined the association between cardiovascular measures and vigilance with the addition of covariates known to impact cardiac metrics.

For within-person associations that were significant for either Black or White individuals, we examined whether the slopes between Black and White individuals were statistically significant in an interaction analysis of the full sample. Covariates for the models were the same as the corresponding race-stratified models. The model examining the two-way interaction between race and daily vigilance predicting negative affect revealed a significant interaction (*b* = -0.09, *SE* = 0.02, *p* < .001) indicating that White individuals (b = 0.14, *SE* = 0.02, *p* < .001) showed a stronger association between within-person vigilance and increased negative affect than Black individuals (*b* = 0.05, *SE* = 0.02, *p* = .003). Examination of the two-way interaction between race and within-person vigilance predicting positive affect revealed a significant interaction (*b* = 0.07, *SE* = 0.03, *p* = .04), indicating that White individuals (*b* = -0.10, *SE* = 0.02, *p* < .001) showed a stronger association between within-person vigilance and decreased positive affect than Black individuals (*b* = -0.03, *SE* = 0.02, *p* = .199). Examination of the two-way interaction between race and within-person vigilance predicting heart rate was non-significant (*b* = 0.12, *SE* = 0.63, *p* = .84). The interaction model predicting heart rate variability was also non-significant (*b* = 0.04, *SE* = 0.02, *p* = .05).

A follow-up analysis removing heart rate values with low reliability (i.e., < 80%) showed similar results and significance for vigilance predicting heart rate (*b* = 0.70, *SE* = 0.30, *p* < .05). The two-way interaction between race and vigilance predicting heart rate while removing heart rate values with reliability less than 80% was not significant. This is consistent with the current study’s analysis.

To provide evidence of validity for our daily measure of vigilance, we examined the baseline vigilance measure’s association with the daily vigilance measure. During a baseline interview, baseline vigilance was assessed through three separate questions with the stem, “How often do you…” and the following items: “...try to prepare for possible insults from other people before leaving home”, “...feel that you always have to be very careful about your appearance to get good service or avoid being harassed”, and “...try to avoid certain social situations and places”. Participants responded using the following scale: 1) At least once a week 2) A few times a month, 3) A few times a year, 4) Less than once a year, and 5) Never. Responses were reverse coded and averaged to create the baseline vigilance score. To examine the association between baseline vigilance and daily vigilance, we used multilevel logistic regression and included the covariates race, age, gender, education, marital status, and depressive symptoms. We found that baseline vigilance significantly predicts daily vigilance (*b* = 0.54, *SE* = 0.23, *p* < .05), providing evidence for the validity of the daily vigilance measure. This measure of daily vigilance is important due to its unique properties that allow for novel within-person associations that we cannot capture with the baseline measure.

To further test the robustness of our findings, we added body mass index (BMI), high blood pressure, number of caffeinated drinks per day, and number of cigarettes smoked per day individually to the models examining vigilance and cardiovascular outcomes. When BMI, high blood pressure, number of caffeinated drinks per day, or cigarettes smoked per day were added individually to the models with vigilance predicting HR and HRV for the whole sample, the direction and significance for vigilance predicting HR or HRV was unchanged. Similarly, for the models with vigilance predicting HR and HRV among White individuals, when BMI, high blood pressure, cigarettes smoked, or caffeinated drinks were added individually to the models the findings were unchanged. Furthermore, in the model with vigilance predicting HR among Black individuals, when BMI, high blood pressure, caffeinated drinks or cigarettes smoked were added the findings were unchanged. In addition, when BMI, high blood pressure, or daily number of caffeinated drinks were added to the model with vigilance predicting HRV among Black individuals, the findings were not changed. However, when the number of cigarettes smoked was added to the model with vigilance predicting HRV among Black individuals, the association between within-person vigilance and HRV became nonsignificant (prior to adding cigarettes, *b* = 0.03, *SE* = 0.02, *p* <.05; after adding cigarettes, *b* = 0.03, *SE* = 0.02, *p* =.06). Notably, the effect estimates and standard errors remained unchanged while the sample size decreased from missing data by 13. This suggests that the nonsignificant finding after adding cigarettes to the model reflects loss of power instead of meaningful attenuation of the association between vigilance and HRV among Black individuals.
